# Modified Nucleoside Triphosphates for *In-vitro* Selection Techniques

**DOI:** 10.3389/fchem.2016.00018

**Published:** 2016-05-04

**Authors:** María A. Dellafiore, Javier M. Montserrat, Adolfo M. Iribarren

**Affiliations:** ^1^Laboratorio de Química de Ácidos Nucleicos, INGEBI (CONICET)Ciudad Autónoma de Buenos Aires, Argentina; ^2^Instituto de Ciencias, Universidad Nacional de General SarmientoLos Polvorines, Argentina; ^3^Laboratorio de Biotransformaciones, Universidad Nacional de QuilmesBernal, Argentina

**Keywords:** SELEX, modified nucleotides, functional oligonucleotides, aptamers, DNAzymes, ribozymes

## Abstract

The development of SELEX (*Selective Enhancement of Ligands by Exponential Enrichment*) provides a powerful tool for the search of functional oligonucleotides with the ability to bind ligands with high affinity and selectivity (aptamers) and for the discovery of nucleic acid sequences with diverse enzymatic activities (ribozymes and DNAzymes). This technique has been extensively applied to the selection of natural DNA or RNA molecules but, in order to improve chemical and structural diversity as well as for particular applications where further chemical or biological stability is necessary, the extension of this strategy to modified oligonucleotides is desirable. Taking into account these needs, this review intends to collect the research carried out during the past years, focusing mainly on the use of modified nucleotides in SELEX and the development of mutant enzymes for broadening nucleoside triphosphates acceptance. In addition, comments regarding the synthesis of modified nucleoside triphosphate will be briefly discussed.

## Introduction

Nowadays, nucleic acids are not only considered as genetic information messengers or repositories. New functions and applications of these molecules, like catalysis and molecular recognition have emerged in the last 25 years (Klussmann, 2006), impacting different fields like: therapeutics (Tei et al., 2015), target validation (Rodríguez et al., 2015), molecular biology (Lee et al., 2015), diagnostics (Wandtke et al., 2015), and analytical chemistry (Li and Lu, 2009; Mascini, 2009; Peinetti et al., 2015). The term “functional oligonucleotide” was coined in reference to these new functions.

Although some examples of these “non-traditional” oligonucleotide activities can be found in nature, as in the case of ribozymes, microRNAs and riboswitches (Li and Lu, 2009), a whole set of catalytic (RNA: ribozymes, DNA: DNAzymes) and molecular recognition oligonucleotides (aptamers) have been synthetically prepared, since they can be obtained using *in vitro* molecular evolution techniques. This methodology merges a combinatorial chemistry approach with a particular property of nucleic acids: amplification with the assistance of polymerases. This procedure was simultaneously developed in three independent laboratories in 1990 (Ellington and Szostak, 1990; Robertson and Joyce, 1990; Tuerk and Gold, 1990) and was denominated SELEX (**S**ystematic **E**volution of **L**igands by **Ex**ponential Enrichment). Since then, many different variations of the technique were developed in order to achieve better selectivity and binding constants, and simpler experimental conditions (Sun and Zu, 2015; Yüce et al., 2015).

During the first years of aptamer development it was soon understood that this functional oligonucleotides could emulate monoclonal antibodies performance in diagnosis (Jayasena, 1999) and therapeutic (Schmid-Kubista et al., 2011) applications. Additionally, aptamers have some advantages over antibodies: they can be chemically synthesized without the assistance of animals; are thermally stable and can be easily fold and unfold. But also they have a major drawback (as DNAzymes and ribozymes); they have short lives in biological fluids due to the ubiquitous presence of endo and exonuclease activities. The strategy to overcome this limitation was the same that was employed in the development of antisense oligonucleotides (Iannitti et al., 2014): the structural chemical modification.

**Figure 1:**
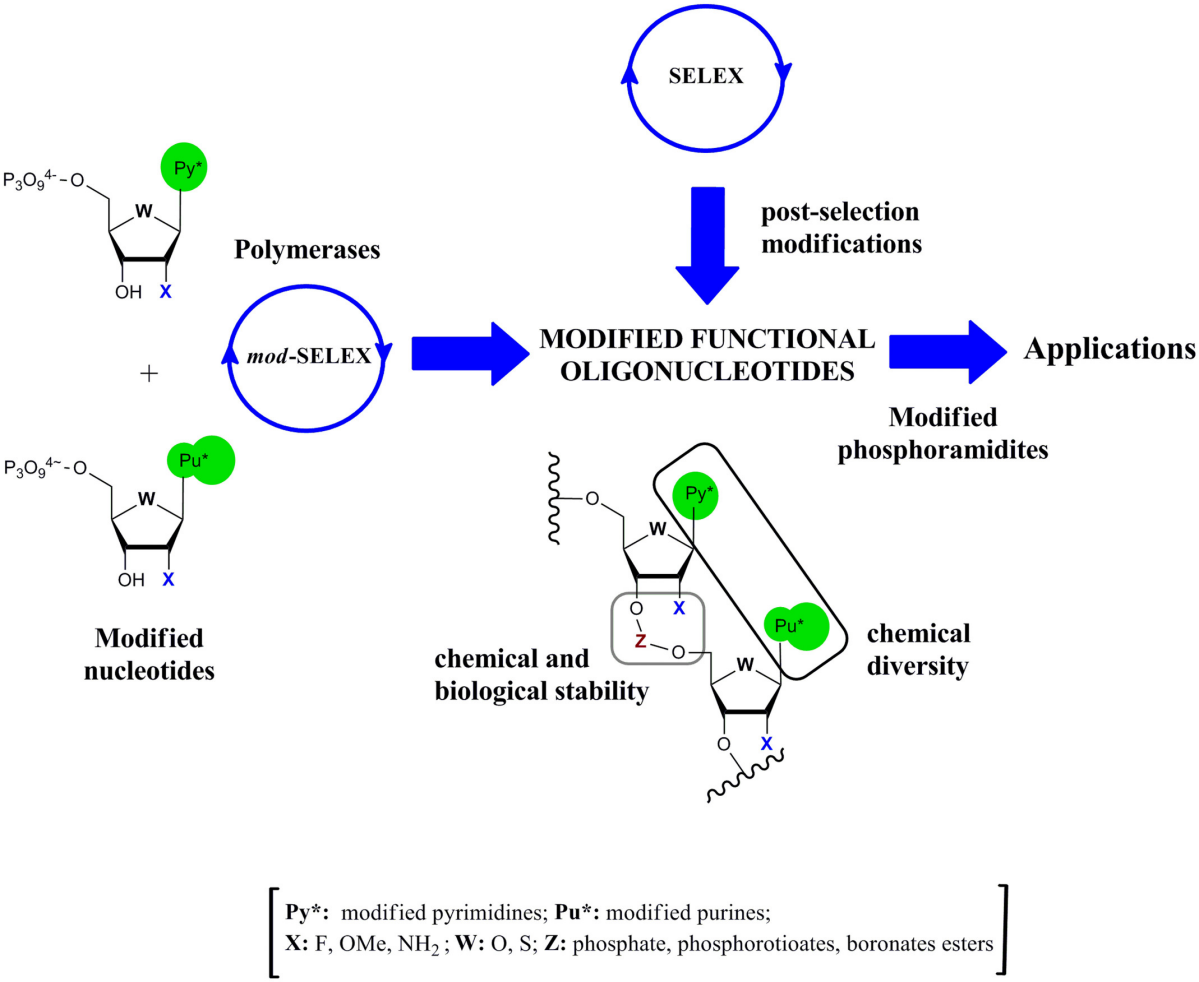
**Schematic representation of the alternative routes to obtain modified functional oligonucleotides**.

Modified functional oligonucleotides could be obtained according to two different strategies: post-selection modification or via modified-SELEX (*mod*-SELEX) techniques (Figure 1). The first approach has the advantage of dealing with natural oligonucleotide chemistry, but as consequence of the delicate relationship between structure and activity, post-selection modification without losing activity demonstrated to be a hard task in ribozymes (Pontiggia et al., 2010), DNAzymes (Robaldo et al., 2014), or aptamers (Bouchard et al., 2010; Förster et al., 2012).

Taking into account this drawback, early efforts were done to modify the SELEX cycle in order to introduce modified nucleotides (Figure 1). The first report of *mod*-SELEX was done by Jayasena group (Lin et al., 1994) who succeeded in performing an *in vitro* selection of an RNA aptamer against human neutrophil elastase (HNE) using 2′-aminopyrimidine nucleotides. The resulting aptamer had a K_*d*_ of (6 ± 3) nM and a half life in serum of (9.3 ± 1.8) h, greatly improving the half life of the unmodified control sequence (4 min). Modifications at the 2′-position of the ribose moiety and phosphate internucleotide linkage were usually introduced in the oligonucleotide structure to enhance the chemical and biological stability (Figure 1).

In addition to stability, another important issue that drives the chemical modification of functional oligonucleotides is the augmentation of structural diversity. In this sense, one of the earliest examples was the obtainment of a DNA anti-thrombin aptamer using 5-pentinyl deoxyuridine by Toole group (Latham et al., 1994). Although the modified aptamer showed a weaker binding constant against thrombin compared to the natural aptamer obtained by the same group (Bock et al., 1992), they showed that the chemical structures of the modified and the natural aptamers were different.

It should be remarked that *mod*-SELEX and post-selection techniques are not excluding strategies as can be learned from the commercial anti-VEGF165 aptamer. Janjic group prepared a 2′-*F*-pyrimidine modified anti-VEGF165 aptamer using a mod-SELEX approach (Ruckman et al., 1998) and after the selection of the best sequence, 2′-*OMe*-ribopurine nucleotides were introduced in some positions without losing binding capacity [K_d_ = (49–130) pM]. This heavily modified aptamer became lately the first example of a therapeutic aptamer approved by the FDA against ocular vascular disease, Pegaptanib (Ng et al., 2006). In a variant of the *mod*-SELEX strategy Mayer and colleagues (Toole et al., 2015) obtained a modified aptamer against cycle 3 GFP (Kd = 18.4 nM) using 5-ethynyl-2′-deoxyuridine triphosphate which was further derivatized after polymerase amplification using click chemistry. A similar strategy, termed SELMA (Selection with Modified Aptamers), was used to generate DNA scaffolds containing ethynyl deoxyuridine moieties that were glycosylated employing glycan azides (Horiya et al., 2014). After selection of the most antigenic clusterings of the glycan, the bound sequences were amplified and reglycosylated to be used in the next selection step.

In the following sections the focus will be set on the different modified triphosphate nucleosides used in *in vitro* selection techniques but it should also be remarked that the modified phosphoramidites (Figure 1) are also essential in order to chemically prepare larger amounts of the modified functional oligonucleotides.

Although comprehensive and recent reviews about the different topics treated in this work are available (Diafa and Hollenstein, 2015; Lapa et al., 2016), the main purpose of this report is to connect all of them having in mind the application of modified nucleotides to *in vitro* selection techniques, focusing on recent related examples.

## Modified nucleotides for SELEX

The design of useful modified nucleotides appropriate for *mod*-SELEX has some restrictions that can be summarized in four conditions (Perrin et al., 1999):

1. should not disturb the base pair interactions (Watson-Crick and Hoogsteen);
2. must be substrates of the corresponding DNA or RNA polymerases;
3. the introduction of the modified nucleotide must be efficient at any position of the sequence;
4. the modified sequence must be a template for the corresponding polymerases.

In addition to the excellent available reviews in this field (Keffe and Cload, 2008; Hollenstein, 2012a; Kong and Bym, 2013) we intend in this section to summarize the main chemical nucleotide modifications that have been used for *in vitro* molecular selection, paying special attention to cases not reviewed earlier.

**Figure 2:**
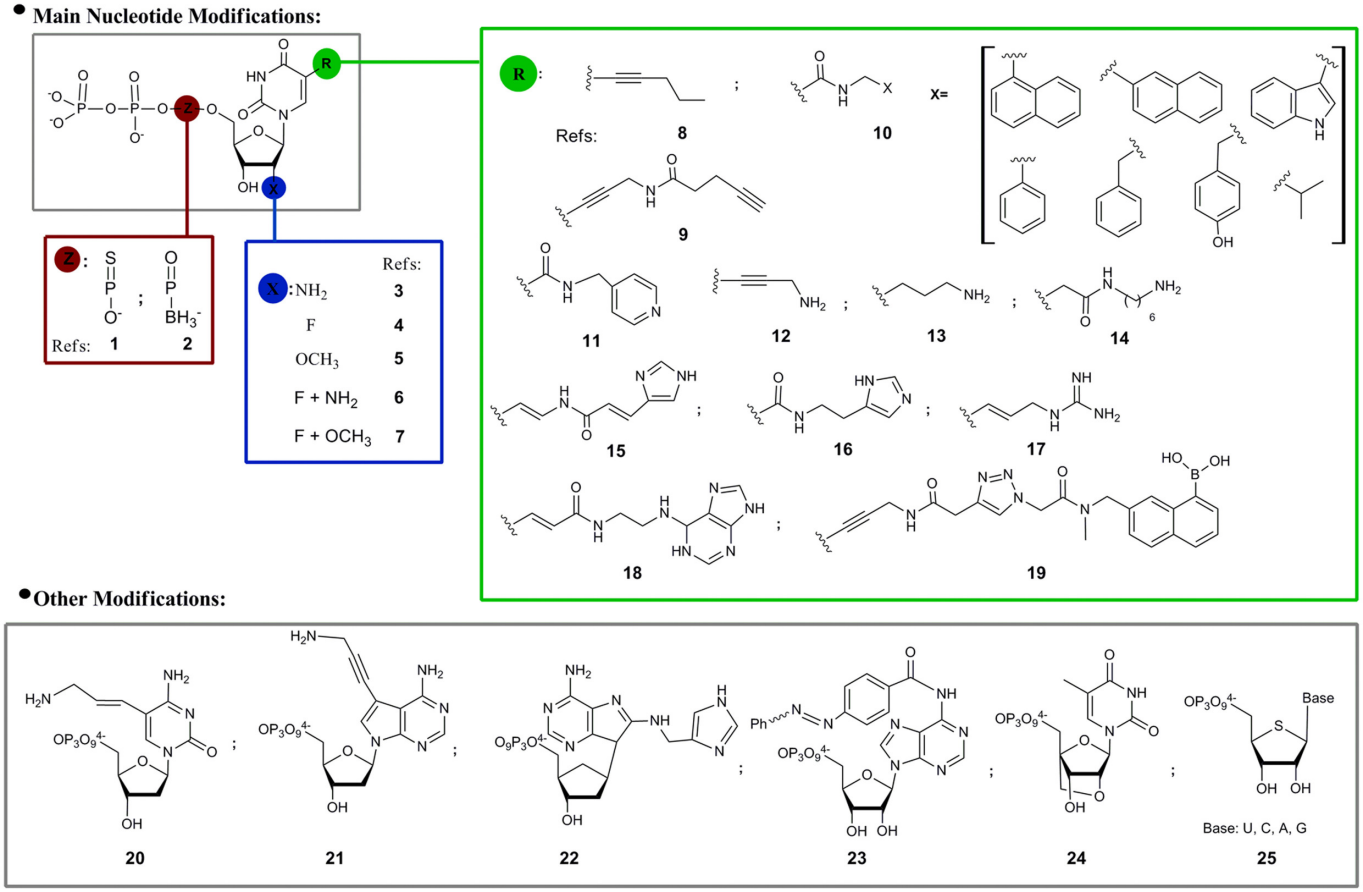
**Modified nucleoside triphosphates used in SELEX experiments**. References: **1**: (Jhaveri et al., 1998; Somasunderam et al., 2010; Higashimoto et al., 2013); **2**: (Lato et al., 2002); **3**: (Lin et al., 1994; Green et al., 1995; Jellinek et al., 1995); **4**: (Svobodova et al., 2013; Dupont et al., 2010); **5**: (Burmeister et al., 2005); **6**: (Pagratis et al., 1997); **7**: (Friedman et al., 2015); **8**: (Latham et al., 1994); **9**: (Li et al., 2008); **10**: (Vaught et al., 2010; Ochsner et al., 2013, 2014); **11**: (Tarasow et al., 1997); **12**: (Battersby et al., 1999); **13**: (Vaish et al., 2003); **14**: (Masud et al., 2004; Shoji et al., 2007); **15**: (Santoro et al., 2000; Sidorov et al., 2004); **16**: (Wiegand et al., 1997); **17**: (Hollenstein et al., 2009); **18**: (Imaizumi et al., 2013); **19**: see 9; **20**: (Hollenstein et al., 2009); **21**: (Sidorov et al., 2004); **22**: (Hollenstein et al., 2009); **23**: (Liu et al., 2010); **24**: (Kasahara et al., 2013); **25**: (Minikawa et al., 2008).

Regarding the modified nucleotides that have been used in SELEX, most examples are related to structural modifications of pyrimidine derivatives in three different positions: the α-phosphate, the 2′- and C5-positions (Figure 2). As consequence of the extraordinary sensitivity to degradation of RNA oligonucleotide libraries, the first *mod*-SELEX examples were motivated by the improvement of oligonucleotide stability against RNases, by substitution of the of 2′-hydroxyl of ribose by other functionalities. As mentioned above, the first reported example was developed by Jayasena group (Lin et al., 1994) who used 2′-aminouridine and 2′-aminocytidine triphosphates (**3**, Figure 2) to obtain a modified RNA aptamer against Human Neutrophil Elastase (HNE). When it was incubated in human serum and human urine, the HNE modified aptamer showed an enhanced stability compared to the unmodified sequence. Although the 2′-aminopyrimidine modification was also used to obtain a modified aptamer against the vascular permeability factor (VPF), the vascular endothelial growth factor (VEGF; Green et al., 1995) and the basic fibroblast growth factor (Jellinek et al., 1995), this modification was no longer used, probably due to the difficulties in the triphosphate preparation and the destabilizing effect of 2-amino groups in RNA duplexes. It was noticed that when the 2′-amino modification was compared to the 2′-fluoro analogs (**4**, Figure 2) for the same target, as in the case of the keratinocyte growth factor aptamer (Pagratis et al., 1997), both modifications have similar nuclease resistance but fluoro analogs have better affinities (two orders). As additional feature, compared to amine groups, 2′-fluoro modification does not require protection-deprotection steps along the oligonucleotide solid phase synthesis, reasons that could partially explain the popularity of this modification (Dupont et al., 2010; Svobodova et al., 2013).

Modified 2′-*O*-methylnucleotides (**5**, Figure 2) have also been used to get an aptamer against VEGF (Burmeister et al., 2005). Keefe and coworkers identified reaction conditions that allowed the incorporation of significant amounts of 2′-*OMe*-deoxyguanosine triphosphate into transcripts in the presence of ribosyl triphosphate. Guanosine positions must be finally checked to confirm the presence of natural or modified nucleotide. The obtained 23 nucleotide long aptamer (ARC245), showed a K_d_ of 2 nM and a fully modified structure which avoids degradation for at least 96 h in plasma. In the same sense, Li group (Friedman et al., 2015) recently developed a fully modified aptamer against *Staphylococcus aureus* Protein A (SpA) using 2′-*F*-deoxyguanosine, 2′-*OMe*-adenosine, -cytidine, and -uridine, an RNA library and a mutant (LAR) T7 RNA polymerase that circumvent the previously mentioned inconvenience of using small amounts of natural ribonucleotides.

A promising field is the aptamer obtainment using nucleotides with fully modified sugar moieties. Chaput and colleagues (Yu et al., 2012) succeeded in the preparation of a Threose Nucleic Acid (TNA) aptamer against human thrombin (Kd = 0.2–0.9 μM) with the assistance of an engineered variant of 9°N DNA polymerase.

Holliger and colleagues (Taylor and Holliger, 2015; Taylor et al., 2015) reported the preparation of four XNAzymes (catalyst from synthetic genetic polymers, XNA) based on four different sugar backbones: Arabino Nucleic Acids (ANA), 2′-Fluoroarabino Nucleic Acids (FANA), Hexitol Nucleic Acids (HNA) and Cyclohexene Nucleic Acids (CeNA). Mutant polymerases D4k for ANA and FANA, 6G12 for CeNA, and 6G12 I512L for HNA were employed for preparing the XNA libraries. Under these conditions RNA endonuclease (ANA, FANA, HNA, CeNA), RNA ligase and XNA ligase (FANA) activities were obtained.

Another position that has been modified with the objective of increasing nuclease resistance is the α-phosphate of nucleoside triphosphates. Ellington and coworkers (Jhaveri et al., 1998) obtained an anti basic fibroblast growth factor (bFGF) RNA aptamer in which all internucleotide linkages were modified with phosphorothioates (**1**, Figure 2). Although the yield of phosphorothiolated RNA following *in vitro* transcription and purification was low, it was enough for selection experiments and a 13 nucleotide consensus sequence with a Kd of 1.8 nM for bFGF was selected. Using the same modification Gorenstein and coworkers (Somasunderam et al., 2010) obtained a thiophosphate-modified DNA aptamer against the hyaluronic acid binding domain (HABD). In this case, all deoxyadenosine residues (with the exception of primers) were replaced by deoxyadenosine thiophosphates in the *S*p configuration. More recently, Yamagishi and coworkers (Higashimoto et al., 2013) selected phosphorothioate-modified aptamers directed against advanced glycation end products (AGEs–thioaptamers) using a mixture of 2′-deoxyadenosine-5′-*O*-(1-thiotriphosphate) (dATP(αS)), 2′-deoxythymidine-5′-*O*-(1-thiotriphosphate) [dTTP(αS)] and a mixture of deoxycytidine and deoxyguanosine triphosphates. The obtained aptamers had K_*d*_ in the low nanomolar range. Another feasible modification in the α-phosphate moiety of the nucleotides is the replacement of oxygen by a borohydride group (boranophosphate, **2**, Figure 2). Burke and coworkers (Lato et al., 2002) explored the use of uridine (bU) or guanosine (bG) 5′-(α-P-borane) in a SELEX process against ATP. Even if bU and bG are compatible with the selection process, when they were introduced in natural aptamer structures usually diminished or eliminated target recognition.

Although the 2′-position and the α-phosphate nucleotide modifications mainly pursue the increase of oligonucleotides stability, another important aspect of SELEX is the expansion of the chemical diversity of the nucleotide moiety. It was reasoned that a set of complementary functional groups, not originally available in the nucleotide structures, will enhance the chances of molecular recognition or catalytic phenomenon. In this regard, 5-position of pyrimidine, particularly uridine and deoxyuridine, was the most frequent modification option. Functional groups present in the 5-pyrimidine position could be classified in three main sets: hydrophobic, acid/base and others (boronic). In the first subset we can find examples such as compounds **8**, **9**, and **10** in Figure 2. Toole and coworkers (Latham et al., 1994) prepared DNA aptamers against thrombin using 5-(1-pentinyl)-2′-deoxyurindine (**8**, Figure 2) with K_d_ in the range of 400–1000 nM. Wang and coworkers (Li et al., 2008) introduced hydrophobic structures (**9**, Figure 2) in aptamers against fibrinogen. In a systematic way, Eaton and coworkers (Vaught et al., 2010) first and Janjic and coworkers later (Ochsner et al., 2014), explored the use of a set of hydrophobic moieties connected to the 5-position of deoxyuridine by an amide group (**10**, Figure 2). In Eaton's work an exploration of the best polymerases that accept the modified nucleotides as substrates was done, finding that the OD XL and D.Vent(exo-) were able to incorporate the modified deoxyuridine triphosphate derivatives with similar or better yields than with thymidine triphosphate. In this case the selected targets were the tumor associated calcium signal transducer 2 (TACSTD2) and the tumor necrosis factor receptor super family member 9 (TNFRSF9). In the first case benzyl and isopropyl moieties were used giving aptamers with K_d_s in the nM range. In the second case, where previous DNA selections failed, the benzyl moiety afforded an aptamer with a K_d_ of 100 nM.

As previously commented, Janjic and coworkers also explored the use of hydrophobic modifications. In this case the objective was to develop methods for the systematic isolation of aptamers that bind to different epitopes of proteins, allowing efficient pair-wise screening of multiple ligands (Ochsner et al., 2014; Rohloff et al., 2014). These aptamers, named slow off-rate modified aptamers (SOMAmer) were prepared using a benzyl moiety linked to the uracil by an amide group and were directed against human proteins like: ANGPT2, TSP2, CRDL1, MATN2, GPVI, C7, and PLG, some of them indicators of cardiovascular risk. The equilibrium dissociation constants ranged from 0.02 to 2.7 nM.

Considering now 5-pyrimidine nucleotide modifications with acid/base activity, a functional group usually present in this position is amine (or ammonium). Eaton and coworkers (Tarasow et al., 1997) synthesized a 5-pyridylmethylcarboxamide uridine (**11**, Figure 2) for the obtainment of a ribozyme with moderate dielsalderase activity (Diels Alder carbon-carbon bond formation). Benner and coworkers (Battersby et al., 1999) synthesized 5-(3″-aminopropynyl)-2′-deoxyuridine triphosphate (**12**, Figure 2) which was used to replace thymidine nucleotide in a SELEX process, with the assistance of the Vent DNA polymerase, against the ATP molecule as target. The consensus sequences were examined to determine if they matched or not the original Szostak sequence (Huizenga and Szostak, 1995), finding significant resemblance with the “Lin-Patel-Huizenga-Szostak motif” (Battersby et al., 1999).

Regarding the existence of modified moieties found in RNA of biological sources, post-transcriptional modifications are implicated in catalytic or molecular recognition events (Limbach et al., 1994; Helm and Alfonzo, 2014). Although moieties with a positive charge are naturally rare, some examples are known, like in the case of archaeosine (Gregson et al., 1993), a modified purine that has a positive charge at physiological pH. This fact inspired McLaughlin and coworkers (Vaish et al., 2003) who used 5-(3-aminopropyl) uridine (**13**, Figure 2) to obtain an RNA aptamer against ATP. The resulting sequence had a K_d_ of 1.08 mM with several of the modified uridines critical for target recognition. Following the same concept, Sawai and coworkers (Masud et al., 2004) used the 5′-triphosphate of 5-*N*-(6-aminohexyl)carbamoylmethyl-2′-deoxyuridine (**14**, Figure 2) for the selection of a DNA aptamer (K_d_ = 4.9 μM) against sialyllactose, an oligosaccharide with a carboxy group that appears to be an essential receptor component of many animal viruses from different families, such as influenza A and C viruses. The same modified pyrimidine was lately used by this research group to develop an aptamer against the (*R*)-isomer of Thalidomide (Shoji et al., 2007) with a K_d_ of 1 μM.

Other nitrogen containing substituents were evaluated. Barbas and coworkers (Santoro et al., 2000) obtained by *in vitro* evolution a DNAzyme with RNase activity carrying an imidazolyl moiety at the 5-position of deoxyuridine (**15**, Figure 2). The DNAzyme has a minimum core of twelve nucleotides including three imidazole-functionalized nucleotides and requires μM concentration of Zn^2+^ and mM of Mg^2+^ and Na^+^ for being active. Lately, Williams and coworkers (Sidorov et al., 2004) used a combination of the 5-imidazolyl-modified deoxy uridine triphosphate (**15**, Figure 2) plus a 7-aminopropynyl modified 7-deaza-deoxyadenosine triphosphate analog (**12**, Figure 2), for the selection of a DNAzyme with RNA cleavage activity independent of divalent metal requirements. Following with the development of modified functional oligonucleotides with catalytic activity, Eaton and coworkers (Wiegand et al., 1997) used 5-imidazolyl uridine triphosphate analog (**16**, Figure 2) for the preparation of a ribozyme with amide-bond formation activity, pushing the hypothesis of the expansion of nucleic acid catalytic activity. With the same objective, Perrin and coworkers (Hollenstein et al., 2009) used a simultaneous combination of 8-histaminyl-deoxyadenosine (**21**, Figure 2), 5-guanidinoallyl-deoxyuridine (**17**, Figure 2) and 5-aminoallyl-deoxycytidine (**20**, Figure 2) triphosphates in an *in vitro* selection of a heavily modified metal^2+^ free DNAzyme with RNase activity. Pursuing a similar goal, Williams and coworkers (Sidorov et al., 2004) prepared and used a 3-(aminopropynyl)-7-deaza-deoxyadenosine triphosphate (**21**, Figure 2). In a recent example, Silverman and colleagues (Zhou et al., 2016) showed that the introduction of protein-like residues (5-thymidine primary alcohol, amine or carboxyl group) allowed the selection of modified DNAzymes with amide hydrolytic activity that was until then an elusive enterprise.

Sugimoto and coworkers (Imaizumi et al., 2013) prepared a (*E*)-5-(2-(N^6^-adeninyl)ethyl))carbamylvinyl)deoxyuridine triphosphate (**23**, Figure 2) that was used to obtain a modified DNA aptamer against camptothecin, a quinoline alkaloid that inhibits topoisomerase I. In this case, the modified aptamer showed a higher affinity constant than the unmodified captothecin aptamer, illustrating the hypothesis that additional aptamer functionalization can improve binding performance.

Returning to modified aptamer examples, Wang and coworkers (Li et al., 2008) prepared a boronic acid 5-modified thymidine triphosphate (**19**, Figure 2) with the aim of improving aptamer binding to glycoproteins (fibrinogen as model), based on the known ability of boron to coordinate glycol groups. The boronic acid modified aptamers selected against fibrinogen had a K_d_ in the low nM range, while aptamers prepared with natural nucleotides had K_d_s ca. 5 μM, confirming the proposed hypothesis.

Ito and coworkers (Liu et al., 2010) prepared a photoresponsive *N*^6^-azobencene-adenosine triphosphate (**23**, Figure 2) for the selection of a modified RNA aptamer against hemin with catalytic activity as peroxidase. The aim of this modification was the photocontrol of the recognition event and consequently of the catalytic activity. The visible and UV irradiation of the obtained modified aptamer controlled the *cis-trans* azobencene isomerization, which modified the hemin-aptamer catalytic ability as peroxidase.

Finally, some modifications in the ribose/deoxyribose moieties have also been explored. Kuwahara and coworkers (Kasahara et al., 2013) explored the use of 2′-*O*, 4′-*C*-methylene-bridged bicyclic ribonucleotides (**24**, Figure 2) for the selection of DNA aptamers against human thrombin. The aptamers were selected by capillary electrophoresis-SELEX (CE-SELEX) and had K_d_ in the low nM range. Matsuda and coworkers (Minikawa et al., 2008) used 4′-thioribonucleotides (**25**, Figure 2) for the selection of an RNA aptamer against human α-thrombin. The optimization of the SELEX conditions for the use of the four modified nucleotides (**25**, Figure 2) included the use of additional amounts of ATP and GTP and the assistance of mutant polymerases. The highly-modified 4′-thioRNA aptamer was also post-selection fully modified, having a K_d_ of 29.6 nM.

**Figure 3:**
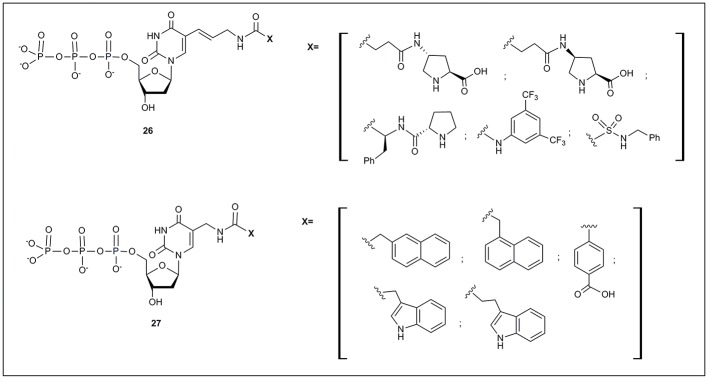
**Modified nucleoside triphosphates accepted by modified polymerases but not yet used in SELEX protocols**.

All the examples of modified nucleotides described so far have been successfully used in SELEX processes finally obtaining functional oligonucleotides. However, some examples of modified nucleotides, accepted by mutant polymerases and not applied yet to SELEX, have also been described. Just to mention two examples, Hollenstein (2012b) has described a set of 5-modified deoxyuridine, with moieties designed for organocatalysis (**26**, Figure 3). These modified nucleotides were substrates of Ven (exo-), Pwo DNA and E. coli Klenow fragment polymerases. More recently, Perrin and coworkers (Liu et al., 2015) reported the preparation of 5-aminomethyl deoxyuridine triphosphate derivatives (**27**, Figure 3) modified mainly with aromatic moieties. The authors successfully used all modified triphosphates as substrates of the Vent (exo-) polymerase.

A mention to L- aptamers (Spiegelmers) will also be included. Although they were not strictly obtained using modified nucleoside triphosphates (Eulberg et al., 2006), the final result of this variant of the SELEX method is a fully modified L-aptamer. This technique has been applied when the enantiomer of the target was synthetically available, as in the case of peptides (Yatime et al., 2015) or RNA structures (Sczepanski and Joyce, 2013).

Another interesting approach is the use of the genetic alphabet expansion. Hirao and colleagues (Kimoto et al., 2013) have described the first example of modified aptamers against human VEGF-165 (Kd = 0.62 nM) and interferon-γ (Kd = 0.038 nM) using the four natural DNA bases plus an additional synthetic base pair in an expanded alphabet strategy, naming the technique as Expanded-SELEX (ExSELEX; Kimoto et al., 2016).

## Modified nucleoside triphosphate synthesis

When modified nucleoside triphosphates (NTPs) for *in vitro* selection techniques are needed they have to be synthesized, since only few of them are commercially available. Therefore, for the safe of completion, a brief summary of the main methods developed for this purpose are herein included.

The regioselective modification of nucleosides is a cumbersome goal due to their polyfunctional structure and in particular an effective and general methodology for the synthesis of NTPs is still a challenge without a proper solution. In addition, NTPs are polycharged and unstable molecules, characteristics that make difficult the work up processes associated with their purification.

The available protocols for NTPs synthesis were extensively reviewed (Burgess and Cook, 2000; Hollenstein, 2012a; Kore and Srinivasan, 2013), and here will be only briefly mentioned.

One of the pioneer methods was the one carried out by Ludwig (Ludwig, 1981) based on the regioselective 5′-phosphorylation designed by Yoshikawa (Yoshikawa et al., 1967). It consists in the one pot reaction of an unprotected nucleoside and phosphorus oxychloride using trimethyl phosphate as solvent. The reactive 5′-phosphorodichlorate intermediate is further treated with bis-tributylammonium pyrophosphate to generate a cyclic triphosphate which is finally hydrolysed to the corresponding NTP. This process is not suitable for modified nucleosides and in addition involves difficult purification processes due to the presence of secondary products. A recent contribution to this strategy (Korhonen et al., 2015) propose the use of tris{bis(triphenylphosphoranylidene) ammonium} (PPN) pyrophosphate as an alternative to the hygroscopic alkylammonium salts.

An alternative method developed by Ludwig and Eckstein (Ludwig and Eckstein, 1989) is still nowadays widely applied for the preparation of NTPs. It is also a one pot route but the nucleoside must be properly protected owing to the lack of regioselectivity of the process. This route involves the attack of salicyl phosphorochlorite to afford the corresponding 5′-phosphite derivative. The subsequent addition of bis-tributylammonium pyrophosphate produces the key cyclic phosphite intermediate, which is then oxidized *in situ* to yield the resultant NTP. Although the interfering nucleosidic functions need to be protected, the purification protocols are simpler since the presence of other polycharged species is minimized.

Another strategy, designed by Caton-Williams et al. (Caton-Williams et al., 2011a,b, 2013), also makes use of the salicyl phosphorochloridite reagent, but through and altered route. In this synthetic scheme the phosphitylating agent is first treated with pyrophosphate in DMF to generate the actual phosphitylating reagent that regioselectively reacts with the 5′-hydroxyl of an unprotected nucleoside to produce a similar cyclic intermediate to the one formed in the Ludwig-Eckstein strategy. Finally, the corresponding NTP is obtained by traditional iodine oxidation and hydrolysis. After performing a simple ethanol precipitation, the crude product can be directly used as polymerases substrate.

Many other different approaches have been explored in the search of a universal and efficient route for the preparation of NTPs, some of them employ nucleoside phosphites, phosphoramidites, diphosphates or displacement of 5′-*O*-leaving groups by triphosphate. A complete revision of these methods has already been reviewed (Burgess and Cook, 2000; Kore and Srinivasan, 2013).

The environmental friendly approaches offered by biocatalysis and biotransformations give an intestering alternative to the chemical preparation of NTPs since they provide regioselective reactions using mild conditions (Staffan, 2005). This process involves three steps being the first one catalyzed by specific nucleoside kinases. Therefore, alternative enzymatic procedures for the preparation of nucleoside monophosphates are relevant and have been recently reviewed (Iglesias et al., 2015).

As for chemical approaches, the biocatalyzed syntheses do not provide a general and efficient method for NTPs preparation and the appropriate route for each case has to be explored specifically.

## Polymerases for modified nucleotides

The increasing number of nucleotide analogs used for the development of functional oligonucleotides demands the access to polymerases with an expanded substrate repertoire. Over the years natural polymerases have been tested for their ability to accept unnatural nucleotides and many directed evolution experiments have been carried out to achieve this goal being these works properly reviewed in several papers (Henry and Romesberg, 2005; Lauridsen et al., 2012; Walsh and Beuning, 2012; Chen and Romesberg, 2014; Laos et al., 2014). A summary of polymerases evolved to accept modified nucleotides can be seen in Table 1.

**Table 1:** **Polymerases evolved to accept modified nucleotides**.

**Polymerase**	**Mutations**	**Activity**	**References**
T7 RNAP (RGFH)	(Y639F)	Incorporates with 2′-F, 2′-amino, 2′-OMe pyrimidines, 2′-deoxy-2′-thio CTP	1;2
T7 RNAP(RGFA)	(Y639F:H784A)	Synthesizes transcripts containing 2′-OMe pyrimidines, 2′-azidoU or 2′-azidoC	3;4;5
T7 RNAP (VRS)	(G542V:H784S:H772R)	Incorporates 2′-F-pyrimidines	5
T7 RNAP (RGVG, E593G, V685A)	(Y639V:H784G:E593G:V685A)	Incorporates 2′-OMe pyrimidines	5
T7 RNAP (RGLH, A255T)	(Y639L:A255T)	Incorporates 2′-F pyrimidines, 2′-OMe pyrimidines	5
Taq DNAP	(I614K)	Incorporates rNTPs more efficiently than wild type	6;7
Taq DNAP	(I614N:L616I)	Incorporates rNTPs more efficiently than wild type	6;7
Taq DNAP	(A661E)	Incorporates rGTPs more efficiently than wild type	8;9
Taq DNAP AA40	(E602V:A608V:I614M:E615G)	RNAP and RT activity. Incorporates rNTP, 2′-azido-NTP and 2′-F-NTP	10
Sf DNAP R1	(K531E:A597T:A600T:W604G:A608S:L609V:I614T:E615G)	Incorporates rNTPs more efficiently than wild type	11
Sf DNAP R2	(A597T:E615G)	Incorporates rNTP substrates more efficiently than wild type	11
Sf DNAP R3	(A597T:W604R:L605Q:I614T:E615G)	Incorporates rNTP substrates more efficiently than wild type	11
Sf DNAP M19	(I614E:E615G)	Incorporates 2′-O-methylNTP, rNTP, 2′-amino-NTP, 2′-azido-NTP, 2′-F-NTP	12;13
Kf DNAP	(I709F)	Incorporates rNTPs more efficiently than wild type	14
TgoT DNAP C7	(TgoT:E654Q:E658Q:K659Q:V661A:E664Q:Q665P:D669A:K671Q:T676K:R709K)	Replication of DNA CeNA and LNA	15
TgoT DNAP D4K	(TgoT:L403P:P657T:E658Q:K659H:Y663H:E664K:D669A:K671N:T676I)	Replication of DNA using ANA, or FANA	15
TgoT DNAP 6G12	(TgoT:V589A:E609K:I610M:K659Q:E664Q:Q665P:R668K:D669Q:K671H:K674R:T676R:A681S:L704P:E730G)	Replication of DNA using HNA	15
TgoT DNAP RT521	(TgoT:E429G:I521L:K726R)	RT activity for oligonucleotides containing HNA, ANA, and FANA	15
TgoT DNAP RT521K	(RT521:A385V:F445L:E664K)	RT activity for oligonucleotides containing CeNA and LNA	15
Taq DNAP M1	(G84A:D144G:K314R:E520G:F598L:A608V:E742G)	Replicates templates containing abasic sites, cis-syn cyclobutane pyrimidine dimer, or 5-nitroindole. Incorporates 7-deaza-dGTP, Rhodamine-5-dUTP, Biotin-16-dUTP, Fluorescein-12-dATP.	16
Taq DNAP	(M444V:P527A:D551E:E832V)	Accepts nucleotides with nonstandard hydrogen bond patterns	17
Taq DNAP	(N580S:L628V:E832V)	Accepts nucleotides with nonstandard hydrogen bond patterns	17
Tth and Taq DNAP chimera 5D4	(V62I:Y78H:T88S:P114Q:P264S:E303V:G389V:E424G:E432G:E602G:A608V:I614M:M761T:M775T)	Forms and extends d5NI and d5NIC self-pairs and heteropairs with all four bases. Extends HBA pairs such as Pyrene: abasic site, d5NI: abasic site, and ICS:7AI	18
Sf DNAP P2	(F598I:I614F:Q489H)	Forms and extends DNA bearing ICS base pairing.	19
Pfu DNAP E10	(Pfu(exo-):V93Q:V337I:E399D:N400D:R407I:Y546H)	Accepts Cy3-dCTP and Cy5-dCTP as substrates.	20
Taq DNAP M1	(G84A:D144G:K314R:E520G:F598L:A608V:E742G)	Accepts phophorothioates. Alllows a full substitution of dNTPs with αS dNTPs.	16
Pfu DNAP	Q484R + split	Incorporates γ-phosphate-O-linker dabcyl derivatives.	21

DNAP, DNA polymerase; RNAP, RNA polymerase; NTP, nucleoside triphosphate; CeNA, cyclohexenyl nucleic acid; LNA, locked nucleic acid; ANA, arabino nucleic acid; FANA, 2′-F-arabino nucleic acid; HNA, 1,5-anhydrohexitol nucleic acid; d5NI, 5-nitroindole; d5NIC, 5-nitroindole-3-carboxamide; ICS, isocarbostyril; 7AI, 7-azaindole. Refrences: 1 (Padilla and Sousa, 1999); 2 (Raines and Gottlieb, 1998); 3 (Padilla and Sousa, 2002); 4 (Burmeister et al., 2006); 5 (Chelliserrykattil and Ellington, 2004); 6 (Patel and Loeb, 2000); 7 (Patel et al., 2001); 8 (Suzuki et al., 1996); 9 (Ogawa et al., 2001); 10 (Ong et al., 2006); 11 (Xia et al., 2002); 12 (Fa et al., 2004); 13 (Schultz et al., 2015); 14 (Shinkai et al., 2001); 15 (Pinheiro et al., 2012); 16 (Ghadessy et al., 2004); 17 (Laos et al., 2013); 18 (Loakes et al., 2009); 19 (Leconte et al., 2005); 20 (Ramsay et al., 2010); 21 (Hansen et al., 2011).

## Polymerases for nucleotides bearing sugar modifications

In the 1990s Aurup et al. demonstrated that wild type T7 RNA polymerase could accept 2′-fluoro-CTP, 2′-fluoro-ATP, 2′-fluoro-UTP, and 2′-amino-UTP as substrates in transcription reactions. Results showed that short templates could be transcribed to full-length RNA products in the presence of these modified nucleotides, although higher affinity was observed for the 2'-amino analogs (Aurup et al., 1992). This enzyme was also found to be capable of synthesizing quimeric nucleic acids composed of ribo and deoxyribonucleotides and of incorporating 2′-*O*-methylnucleotides in various positions (Conrad et al., 1995).

Since then, many directed evolution experiments have been performed to improve T7 RNA polymerase substrate repertoire and processivity. Rational design led to a T7 RNA polymerase bearing the Y639F mutation, which was able to synthesize transcripts with 2′-fluoro, 2′-amino, 2′-*O*-methylpyrimidines and 2′-deoxy-2′-thio-CTP more efficiently than the wild type (Raines and Gottlieb, 1998; Padilla and Sousa, 1999). A double mutant (Y639F:H784A) was later reported that showed improved use of 2′-*O*-methyl and 2′-azido pyrimidines as substrates (Padilla and Sousa, 2002). Using the autogene selection method many other variants were reported with better utilization of 2′-substitued NTPs (Chelliserrykattil and Ellington, 2004). Interestingly, mutations previously shown to increase the thermal tolerance of T7 RNA polymerase can also increase the activity of mutants with expanded substrate range (Meyer et al., 2015). T7 RNA polymerase was also tested for its ability to accept 2′-*C*-branched NTPs. Wild type enzyme was able to perform the reaction with 2′-α-hydroxymethyl-UTP while the Y639F mutant was unable to use any of the substrates (Pavey et al., 2004). In another work, the wild type enzyme was capable of synthesizing 4′-thioRNA using 4′-thioUTP and 4′-thioCTP (Kato et al., 2005).

Regarding accessible enzymes, it is worth mentioning that many commercially available DNA polymerases such as SequiTherm, Tht, AmpliTaq FS (exo-), Pfu (exo-), Vent (exo-), and deep Vent (exo-) as well as the human DNA polymerases α and γ, were qualified to accept 2′-fluoro-modified NTPs (Ono et al., 1997; Richardson et al., 2000).

Directed evolution efforts have also focused on the widely used Taq DNA polymerase to improve 2′-modified nucleotides recognition. Several variants of this enzyme have been identified to incorporate ribonucleotides more efficiently than the wild type (Suzuki et al., 1996; Patel and Loeb, 2000; Ogawa et al., 2001; Patel et al., 2001) whereas Taq DNAP AA40 was able to incorporate ribonucleotides as well as 2′-azido and 2′-fluoro NTPs (Ong et al., 2006). Stoffel fragment (Sf) DNA polymerase, a proteolytic fragment of Taq DNA polymerase, was also subjected to directed evolution obtaining the variants Sf R1, Sf R2 and Sf R3 with an increased efficiency to incorporate ribonucleotides (Xia et al., 2002), and Sf M19 that could also accept 2′-*O*-methyl NTP, 2′-amino-NTP, 2′-azido-NTP and 2′-*F*-NTP as substrates (Fa et al., 2004). A recent study improved the understanding of the mutational origins of 2′-modified substrate recognition, and also identified Sf M19 as the best candidate for further engineering (Schultz et al., 2015). The Klenow fragment (Kf), a proteolytic fragment of *E. coli* DNA polymerase I, bearing one mutation (I709F), presented better incorporation of ribonucleotides to the product than the wild type (Shinkai et al., 2001).

Another interesting finding regarding 2′-modified nucleotides was the ability to add 2′-C-methyl analogs into RNA by a hepatitis C virus RNA polymerase. Hepatitis C virus nonstructural protein 5B (HCV NS5B) accepted 2′-C-methyl-ATP and 2′-C-methyl-CTP as substrates while mutant NS5BΔ55 incorporated the modified nucleotides more efficiently (Carroll et al., 2003; Dutartre et al., 2006).

Enzymatic polymerization using LNA nucleotides was reported using many different polymerases. First, the group of Wengel (Veedu et al., 2007) reported that Phusion High-Fidelity DNA polymerase had the capacity of adding up to three LNA-TTPs and up to eight consecutive LNA-ATPs. Later, results showed that 9°NmTM DNA polymerase presented this activity as well, and was able to read across LNA residues in the template. In addition, T7 RNA polymerase was capable of transcription incorporating LNA-ATPs as substrates and also full length RNA transcripts were obtained when LNA and DNA templates were involved (Veedu et al., 2008). In another work, it was observed that using higher concentrations of KOD Dash, KOD (exo-) and Vent (exo-) DNA polymerases led to higher yields of full length products bearing LNA modifications (Kuwahara et al., 2008).

Several DNA polymerases were tested for their ability to synthesize 2′-deoxy-2′-fluoro-β-D-arabino nucleic acids (FANA). Experiments revealed that family B polymerases like Deep Vent (exo-), 9°Nm, Therminator and Phusion High-Fidelity were able to incorporate all four FANA analogs to yield full length products (Peng and Damha, 2007). A later investigation, proposed the development of polymerases that could synthesize Xeno nucleic acids (XNA, nucleic acids carrying different types of synthetic sugars) from a DNA template and polymerases that could reverse transcribe XNA back into DNA. Using compartmentalized self-tagging (CST) selection from a library of TgoT DNA polymerase, they obtained TgoT mutants that were able to replicate DNA using cyclohexenyl nucleic acids (CeNA), locked nucleic acids (LNA), arabino nucleic acids (ANA), 2′-fluoroarabinonucleic acids (FANA) and 1,5-anhydrohexitol nucleic acids (HNA) as substrates. By saturation mutagenesis based on statistical correlation analysis (SCA) and ELISA-like screening they evolved TgoT into variants capable of reverse transcription of templates containing HNA, ANA, FANA CeNA, and LNA (Pinheiro et al., 2012).

Recently, Holliger and co-workers (Cozens et al., 2015) engineered the polymerase TGLLK (TgoT: Y409G, I521L, F545L, E664K), which proved to be effective at fully replacing purine dNTPs and NTPs with their respective 3′-deoxy or 3′-*O*-methyl analogs with defined 2′-5′ linkages allowing template-directed synthesis and reverse transcription of oligonucleotides with mixed 2′-5′/3′-5′ backbone linkages, expanding the repertoire for future *in vitro* evolution experiments.

## Polymerases for nucleotides bearing nucleobase modifications

Different thermostable DNA polymerases were tested using C5-substituted pyrimidines as substrates. Family A DNA polymerases, such as Taq, Tht and Thermosequenase, were able to accept propynyl dUTP, dUTP, and methyl dCTP whereas family B DNA polymerases, such as Pwo, Pfu, Vent (exo-), Deep Vent (exo-), were able to incorporate 7-amino-2,5-dioxaheptyl dUTP, 7-amino-2,5-dioxaheptyl dCTP, propynyl dUTP, and methyl dCTP (Kuwahara et al., 2003). Vent (exo-), Pwo and Kf DNA polymerases were also able to use NTPs carrying urea, proline, and sulfonamide groups in the C5 position (Hollenstein, 2012b). DNA molecules containing 5-vinyl-dUTP, 5-vinyl-dCTP, or 7-deaza-7-vinyl-dATP were prepared by polymerase incorporation using Pwo, Vent (exo-), and KOD XL DNA polymerases (Mačková et al., 2014).

KOD Dash DNA polymerase was used to perform SELEX of sialyllactose-binding DNA aptamers composed of several modified TTPs bearing a positively-charged amino group at the C5 position (Masud et al., 2004). Later, it was also showed the ability to successfully accept dUTP bearing different aminoacids at the C5 position (Kuwahara et al., 2006). Useful dCTP analogs for aptamer discovery via SELEX bearing a 5-(N-substituted-carboxamide) functional group were found to be suitable substrates of KOD DNA polymerase (Rohloff et al., 2015).

Directed evolution of Taq DNA polymerase led to more efficient enzymes that could bypassed blocking lesions such as an abasic site, a thymidine dimer or the base analog 5-nitroindol (Ghadessy et al., 2004; Loakes et al., 2009), and variants that could accept nucleotides with nonstandard hydrogen bond patterns therefore allowing the expansion of the genetic alphabet (Laos et al., 2013).

Using directed short-patch compartmentalized self-replication (spCSR) and the widely used fluorescent dye label Cy3-dCTP and Cy5- dCTP as substrates, a variant of Pfu DNA polymerase that was able to amplify double stranded DNA fragments incorporating these analogs was obtained (Ramsay et al., 2010).

Recent studies (Wyss et al., 2015) involving the ability to amplify DNA adducts showed that a mutant of KlenTaq DNA polymerase (KTqM747K), was able to incorporate an artificial nucleotide, BenziTP, opposite to a DNA alkylation adduct with high selectivity. In this way, the artificial nucleotide functions as a marker for the adduct in the original template being useful to investigate DNA damage levels.

## Polymerases for nucleotides bearing triphosphate modifications

Nucleosides carrying 5′-*O*-(1-thiotriphosphate) moieties (NTP(αS)) are the most employed triphosphate modified substrates for natural and evolved enzymes used for the selection of aptamers. Taq DNA polymerase has been used to select a single-stranded DNA aptamer targeted to the transforming growth factor-b1 (TGF-b1) where all dATPs and dCTPs were substituted by phosphorothioates (Kang et al., 2008). In another study, the M1 variant of Taq DNA polymerase allowed the full substitution of dNTPs with phosphorothioates (Ghadessy et al., 2004). Regarding less frequent modifications, T7 RNA polymerase was used to select an ATP-binding aptamer containing 5′-(α-P-borano)-GTP and 5′-(α-P-borano)-UTP (Lato et al., 2002) and a mutated Pfu DNA polymerase was able to incorporate nucleotides bearing a bulky γ-phosphate-O-linker-dabcyl substituent (Hansen et al., 2011).

## Conclusions and perspectives

The vast number and types of applications that modified functional oligonucleotides offer to different fields such as diagnosis, therapy, analytical chemistry, target validation and molecular biology, makes of the use of modified nucleoside triphosphates for *in vitro* selection techniques a challenging research area. This strategy brings about a second generation of functional oligonucleotides with higher nuclease resistance and increased structural and chemical diversity allowing the selection of molecules with differential properties respect to the natural DNA or RNA oligomers such as improved binding or catalytic activities. In addition, other benefits such as better uptake for *in vivo* applications are expected.

Still many contributions and new developments are needed mainly from two disciplines: molecular biology and organic chemistry. The first one should generate a larger repertoire of evolved polymerases able to faithfully recognize a wider spectrum of modified nucleoside triphosphates. On the other side, although chemistry already made available a plethora of modified nucleosides, it still needs to provide a general and efficient synthetic route to modified nucleoside triphosphates and to propose improvements of the current complex purification protocols.

The research carried out in these areas, and summarized in this review, indicates that in the near future these issues will be successfully addressed expanding the scope of functional oligonucleotides as useful custom-made tools for *in vivo* and *in vitro* applications.

## Author contributions

JM mainly contributed to write the modified nucleotides for SELEX and introduction sections. MD mainly contributed to review the Polymerases for modified nucleotides section. AI mainly contributed to write the modified nucleoside triphosphate synthesis and Conclusions and perspectives sections and revised the whole paper.

## Funding

JM and AI are research members of CONICET and MD has a doctoral fellowship from CONICET. This work has been partially supported by PICT 2011–2007.

### Conflict of interest statement

The authors declare that the research was conducted in the absence of any commercial or financial relationships that could be construed as a potential conflict of interest.
